# Low Temperature NH_3_-SCR over Mn-Ce Oxides Supported on MCM-41 from Diatomite

**DOI:** 10.3390/ma12223654

**Published:** 2019-11-06

**Authors:** Mingxuan Ma, Xiaoyu Ma, Suping Cui, Tingting Liu, Yingliang Tian, Yali Wang

**Affiliations:** College of Materials Science and Engineering, Beijing University of Technology, Pingleyuan 100, Chaoyang District, Beijing 100124, China; mmx0723@outlook.com (M.M.); ltt15063292858@163.com (T.L.); tianyl@bjut.edu.cn (Y.T.); wangyali1978@bjut.edu.cn (Y.W.)

**Keywords:** diatomite, molecular sieve, MCM-41, denitration catalyst, SCR

## Abstract

A series of MCM-41 molecular sieves with different molar ratio of template to silicon were synthesized through hydrothermal synthesis method by using cetyltrimethylammonium bromide (CTAB) as the template, diatomite as the silicon source. By using impregnation method, the Mn-Ce/MCM-41 SCR molecular sieve-based catalysts were prepared. The results observed that when the molar ratio of template to silicon was 0.2:1, the MCM-41 as catalyst carrier has the highest surface area and largest pore volume, it also presented typically ordered hexagonal arrays of uniform channels. The denitration catalytic material based on this carrier has a high number of Lewis acidic sites, and the denitration efficiency can reach more than 93%.

## 1. Introduction

Nitrogen oxides (NO_x_) is a general term for various nitrogen oxides including NO, NO_2_, and N_2_O. As a major source for air pollution, nitrogen oxides contribute to acid rain [1], photochemical smog [2], and ozone depletion [3]. Selective catalytic reduction (SCR) of NO_x_ by ammonia (NH_3_) is a widely used technology for reducing nitrogen oxides emissions from stationary sources, it uses ammonia (NH_3_) as a reducing agent and decomposes NO_x_ among the flue gas into harmless N_2_ and H_2_O through the use of catalyst [4]:

(1) $$4\mathrm{NO}+4{\mathrm{NH}}_{3}+O_{2}=4N_{2}+6H_{2}O$$

Undesirable processes occurring in SCR systems include several competitive, nonselective reactions with oxygen, which is abundant in the system. These reactions can produce secondary emissions:

(2) $$2{\mathrm{NH}}_{3}+2O_{2}=N_{2}O+3H_{2}O$$

(3) $$4{\mathrm{NH}}_{3}+5O_{2}=4\mathrm{NO}+6H_{2}O$$

For low-temperature (<300 °C) SCR catalysts, current research had focused on TiO_2_-supported manganese oxides catalyst. Manganese oxides contained various types of labile oxygen which were believed to play an important role in the SCR catalytic circle. CeO_2_ could enhance the oxidation of NO to NO_2_, which were favorable to the low-temperature SCR of NO with NH_3_, because of its redox properties [5,6]. But titanium dioxide is expensive and has a relatively low specific surface area [7]. Therefore, it is of great significance to study the use of other materials instead of titanium dioxide as a catalyst carrier to prepare an inexpensive and high-performance denitration catalyst.

MCM-41 molecular sieve was originally synthesized and proposed by Beck from Mobil Oil Corporation in the early 1990s [8]. MCM-41 can be synthesized using organic silicon source with an organic surfactant as template. MCM-41 molecular sieve has the advantages of large specific surface area, narrow pore size distribution, high thermal stability and regular arrangement of tubular pores in hexagonal structured network [9]. Because of its large specific area, it can be used as a catalyst carrier to support active components such as metal oxides [10]. 

Diatomite is a powdery, non-metallic mineral composed of the fossilized skeletal remains of diatoms which are microscopic single-celled aquatic plants. The main component of diatomite is SiO_2_, which also contains a small amount of Al_2_O_3_ and Fe_2_O_3_, SiO_2_ content of diatomite can reach more than 80%. The interior of diatomite has many nano-scale microporous structures and has strong adsorption capacity. Currently, it has been widely used for filter aids and water treatment. China’s diatomite reserves are almost 390 million tons. Because of its high silicon content and its low price, it could be used as an ideal raw material for pure silica MCM-41 [11].

In this study, diatomite was used as silicon source instead of the conventionally used organic silicon source such as tetraethyl orthosilicate (TEOS). MCM-41 molecular sieve was synthesized by hydrothermal method using cetyltrimethylammonium bromide (CTAB) as a template. By using impregnation method, the metal oxide of manganese and cerium as active component, denitration catalyst were synthesized. The effects of different molar ratio of template to silicon on the structure and morphology of MCM-41 were investigated, and denitration catalytic properties of MCM-41 supported Mn-Ce catalyst were studied.

## 2. Materials and Methods

### 2.1. Catalyst Preparation

**Figure 1:**
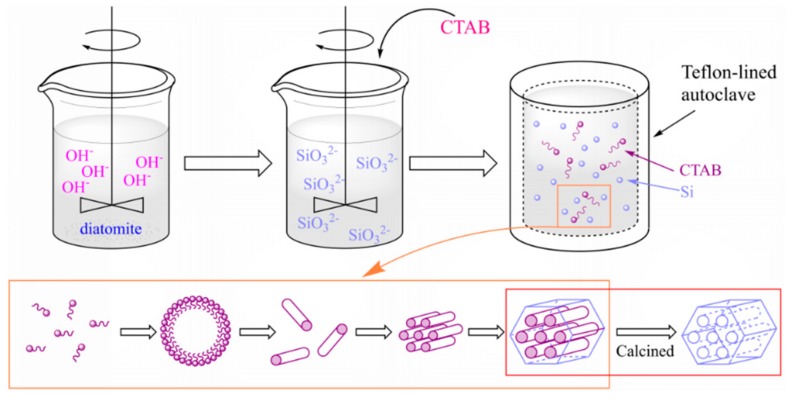
Schematic representation of MCM-41 synthesis.

The synthesis route of MCM-41 molecular sieve is shown in Figure 1. The diatomite used in this study was produced in Changbai, Jilin, China. Cetyltrimethylammonium bromide (CTAB) was used as template and diatomite as silicon source. The diatomite was mixed with 250 mL NaOH (1.5 mol/L) and heated to 90 °C and stirred at a speed of 200 rpm for 2 h. The sodium silicate solution was obtained through filtration.

The filter residues were washed with deionized water and dried at 60 °C overnight, then the filter residues were weighed and subjected to XRF analysis. The results are shown in Table 1, the filter residues 1 and 2 were derived from two different batch reaction. By calculating the difference of Si content between diatomite and the filter residues, the silicon content in the filtrate was about 0.32 mol/L. CTAB (2.33 g, 4.66 g, 6.99 g) was dissolved in 100 mL deionized water by the ultrasound method (45 KHz, 40 °C). After it is completely dissolved, it is mixed with 200 mL filtrate to make n(CTAB):n(Si) = 0.1:1, 0.2:1, 0.3:1, regulated pH value to 9. After stirring at a speed of 250 rpm for 80 min, the obtained mixture was sealed in a Teflon-lined autoclave for hydrothermal treatment at 100 °C for 24 h. The solid product was obtained through filtering, washing with deionized water, and dried at 60 °C overnight. Finally, the samples were grinded down, heated to 550 °C at 2 °C /min in air and calcined for 5 h to obtained MCM-41. The prepared samples were represented by MCM-41(x), where x was the molar ratio of CTAB to silicon, n(CTAB):n(Si) = x:1.

**Figure 2:**
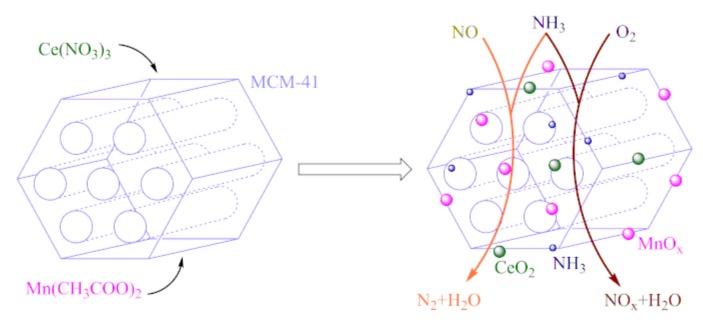
Schematic representation of catalyst preparation and SCR reaction.

The preparation of catalysts is shown in Figure 2. Mn-Ce/MCM-41 catalysts were prepared by impregnation method. Manganese acetate was used as a precursor of MnO_x_, and cerium nitrate was used as a precursor of CeO_2_. For Mn-Ce/MCM-41 catalysts, 3 g of MCM-41 was added as the catalyst support into 150 mL aqueous solution of Ce(NO_3_)_3_·6H_2_O and Mn(CH_3_COO)_2_·4H_2_O, the molar ratio of Mn/Ce/Si was 15:5:100. The obtained mixture was stirred for 24 h at room temperature, then the mixture was filtrated and evaporated at 60 °C to dryness. The solid sample was ground and heated to 450 °C at 5 °C /min in air, calcined for 4 h to obtained Mn-Ce/MCM-41 catalysts. The prepared samples were represented by Mn-Ce/MCM-41(x), where x was the molar ratio of CTAB to silicon when the MCM-41 carriers was prepared. The same impregnation process was carried out on the diatomite, and the same active component was loaded to prepared a denitration catalyst using diatomite as a carrier, which was recorded as Mn-Ce/diatomite.

### 2.2. Characterization Techniques

Specific surface area and pore structure analysis of MCM-41 molecular sieves and MCM-41 catalysts were determined by N_2_ adsorption/desorption isotherms recorded at −195.8 °C with a Micromeritics’s TriStar II 3020 high speed automatic specific surface area and pore analyzer (Micromeritics, Norcross, GA, USA). The specific surface areas and pore size distributions were assessed by BET method and BJH method. Before measurement, 0.2–0.3 g samples were ground to 100 mesh degassed under vacuum at 350 °C for 4 h.

Elemental chemical analysis of diatomite and MCM-41 catalysts was performed by X-ray fluorescence spectrometry (XRF) on a Shimadzu’s XRF-1800 sequential X-ray fluorescence spectrometer (Shimadzu, Kyoto, Japan). Before analysis, all powder samples were dried at 80 °C for 1 hour and tabulated to 30 mm diameter samples.

For each test, the powder samples were ground to 100 mesh and dried at 80 °C for 1 h. The powder X-ray diffraction (XRD) measurement was used to analyze the structure of the MCM-41 with XRD-7000 X-ray diffraction analyzer produced by Shimadzu Corporation (Kyoto, Japan), using Cu Kα radiation (40 kV, 30 mA). Infrared spectra were recorded by a Bruker TENSOR 27 FT-IR spectrometer (Bruker, Billerica, MA, USA) with a Bruker Platinum ATR attachment (Bruker, Billerica, MA, USA). The SU8020 scanning electron microscope (SEM) of Hitachi company (Tokyo, Japan) was used for analyzing the microstructure of the MCM-41. The mesoporous structure of sample was observed by a JEM-2100F transmission electron microscopy (TEM) (JEOL, Akishima, Japan). The temperature programmed desorption (NH_3_-TPD) was performed on Chembet Pulsar TPR/TPD automatic chemisorption analyzer (Quantachrome Instruments, Boynton Beach, FL, USA). The in situ DRIFT experiments were performed on a Bruker Vertex70 with a Pike DiffusIR cell attachment (PIKE Technologies, Fitchburg, WI, USA).

The SCR activity measurements were carried out in a fixed-bed quartz reactor (inner diameter = 30 mm) by using 2 mL catalyst with 20–60 mesh. The reactant-gas composition is as follows: 1000 ppm NO, 1000 ppm NH_3_ and 5 vol % O_2_ balanced by N_2_ with a total flow rate of 900 mL/min and a GHSV of 27,000 h^−1^. For each test, the catalyst was stabilized for 15 min to reach the steady state at a given temperature before measuring its denitration activity. The compositions of the outlet gas were analyzed by a Bruker TENSOR 27 FT-IR spectrometer with a PIKE 162-20XX Heated Gas Flow Cell attachment (PIKE Technologies, Fitchburg, WI, USA). The denitration rate and N_2_ selectivity were calculated as following formula:

(4) $${\mathrm{NO}}_{x}\;\mathrm{conversion}\left(\%\right)\;=\;\frac{[\mathrm{NO}{]}_{\mathrm{in}}\;-\;[\mathrm{NO}+{\mathrm{NO}}_{2}{]}_{\mathrm{out}}}{{\left[\mathrm{NO}\right]}_{\mathrm{in}}}\;\times \;100$$

(5) $$N_{2}\;\mathrm{selectivity}\left(\%\right)\;=\frac{\left({\left[\mathrm{NO}\right]}_{\mathrm{in}}\;+{\left[{\mathrm{NH}}_{3}\right]}_{\mathrm{in}}\right)\;-{\left[{\mathrm{NO}}_{2}\right]}_{\mathrm{out}}\;-\;2{\left[N_{2}O\right]}_{\mathrm{out}}}{{\left[\mathrm{NO}\right]}_{\mathrm{in}}\;+{\left[{\mathrm{NH}}_{3}\right]}_{\mathrm{in}}}\times \;100$$

## 3. Results and Discussion

### 3.1. Analysis of Composition and Morphology of Diatomite

**Figure 3:**
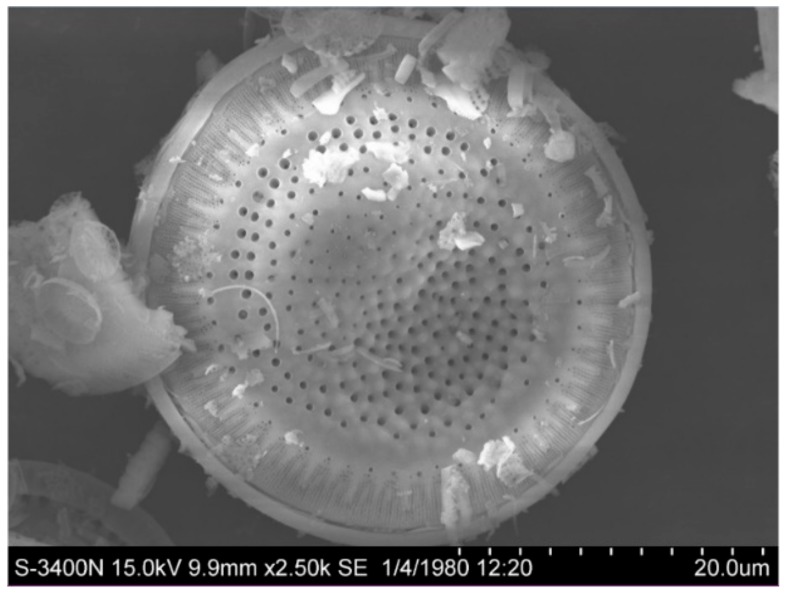
Scanning electron microscope (SEM) image of diatomite.

The diatomite was ground and dried in 60 °C for 24 h to obtain a white grey powder. The chemical composition was analyzed by X-ray fluorescence spectrometer. The results are shown in Table 2. The silica content in the diatomite is 95.36%, the Fe_2_O_3_ content is 1.41%, the Al_2_O_3_ content is 1.08%, the K_2_O content is 0.86%, and impurities such as CaO content and Na_2_O content are also contained. It can be seen that diatomite has a high silicon content and can be used as an ideal raw material for the synthesis of MCM-41 molecular sieve. Figure 3 is an SEM image of the diatomite, it shows that it has typical diatom structural characteristics. A single diatomite particle is about 35 μm in diameter with a certain number of nanoscale channels on the surface.

### 3.2. Specific Surface Area and Pore-size of MCM-41 Catalyst Carrier and Diatomite

For the catalytic material, in general, the larger the specific surface area and pore volume, the larger the contact area of the reaction gas with the catalytic material, the smaller the internal diffusion resistance of the reaction gas on the surface of the catalytic material, and the faster the reaction rate [12]. It can be seen from Table 3 that the specific surface area of each of the MCM-41 carriers is above 800 m^2^/g, and the specific surface area and pore volume thereof are greatly improved compare with the diatomite. The MCM-41(0.2) has the largest specific surface area, reaching 941 m^2^/g, and its average pore volume is 0.94 cm^3^/g. The three MCM-41 carrier pore sizes are all around 3.4 nm.

**Figure 4:**
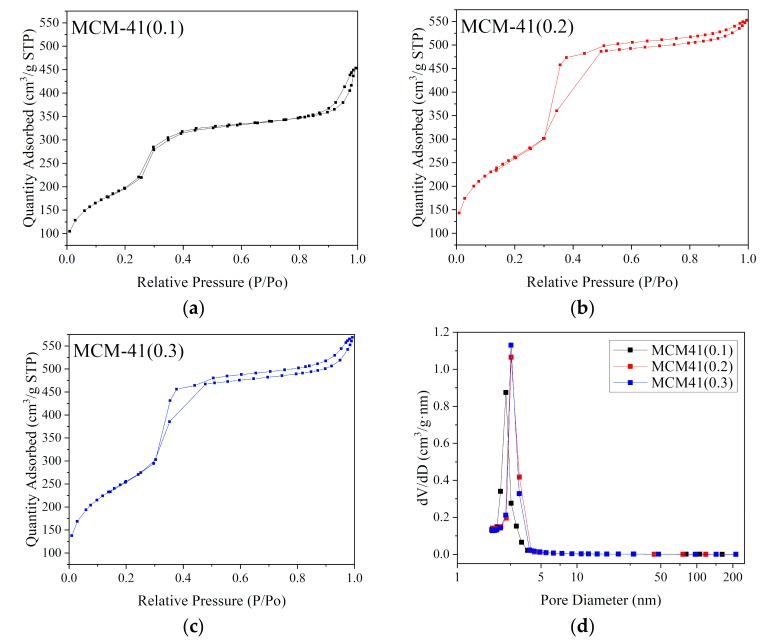
N_2_ adsorption-desorption isotherms (**a**–**c**) and pore diameter distribution curves (**d**) of MCM-41(x).

The nitrogen adsorption-desorption isotherms of MCM-41 samples are shown in Figure 4a–c and resembles type IV isotherms because of capillary condensation within uniform mesopore channels which is a characteristic behavior of the mesoporous structure. The pore diameter distributions in Figure 4d were derived from BJH adsorption curves. The N_2_ isotherms of 3 MCM-41 samples exhibit a sharp increase in N_2_ volumes adsorbed at P/P_0_ = 0.3–0.5. A hysteresis loop which is corresponding to a wide area in MCM-41(0.2), MCM-41(0.3) can be observed at P/P_0_ = 0.3–0.5, indicates that MCM-41(0.2) and MCM-41(0.3) has a larger specific surface area compare to MCM-41(0.1). For MCM-41(0.1), the adsorption isotherms of MCM-41(0.1) features a very narrow hysteresis loop, which indicates a very narrow pore size distribution. This is very consistent with the pore size distribution data in Figure 4d. The MCM-41(0.1) exhibits a hysteresis loop at the P/P_0_ close to 1, indicating the existence of the interparticle pores.

### 3.3. FT-IR Analysis of MCM-41

**Figure 5:**
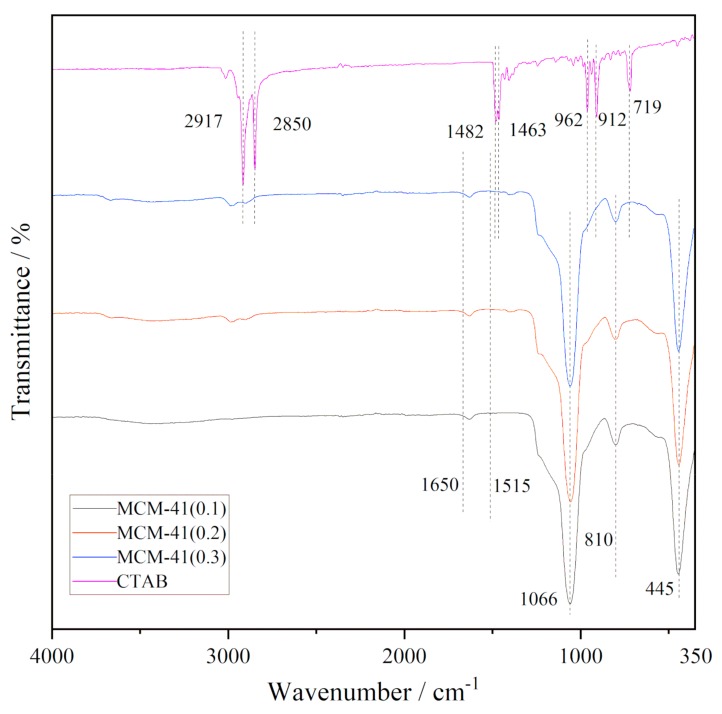
The FT-IR spectra of CTAB and MCM-41(x).

Figure 5 shows the IR spectra of the CTAB and support MCM-41. In the IR spectrum of CTAB (Aladdin, 99%), the CH_2_ symmetric and asymmetric stretching vibrations lie at 2917 and 2850 cm^−1^ [13]. The absorption peak at 1482 and 1463 cm^−1^ can be assigned to C–H scissoring vibration of CH_3_–N^+^ moiety [13] and deformation bending vibration of alkyl chain [14]. The peaks at 962 and 912 cm^−1^ belong to C–N stretching bands, the peak at 719 cm^−1^ belongs to rocking mode of the methylene chain [13].

The MCM-41 supports have three obvious infrared absorption peaks at different positions, of which the absorption peak at 1066 cm^−1^ can be assigned to an asymmetric stretching of the Si-O bond, and the absorption peaks at 810 cm^−1^ and 445 cm^−1^ are associated with a symmetric stretching and bending of the Si–O bond [15]. The absorption peaks associated with CTAB could not be found in the spectra of MCM-41(x). Furthermore, the peak at 1650 cm^-1^ assigned to C=C bonds and 1515 cm^−1^ attributed to C–C vibration corresponding to the amorphous or graphitic carbon [16] do not appear at the spectra of MCM-41(x), which indicates that the carbon species in MCM-41 supports are below the detection limit of FT-IR, therefore the template has been totally removed after the calcination step.

### 3.4. XRD Analysis of MCM-41

**Figure 6:**
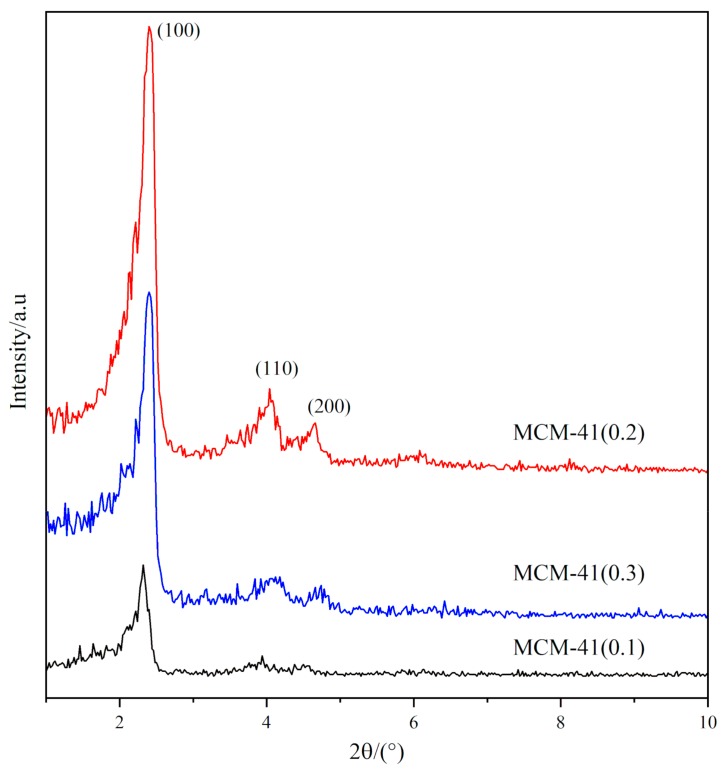
XRD patterns of MCM-41 with different ratio of template.

In order to further analyze the structure of MCM-41 molecular sieve catalyst carrier, different samples were analyzed by XRD, and the results are shown in Figure 6. The XRD patterns reveal the regular structure of the MCM-41, for MCM-41(0.2) and MCM-41(0.3), one prominent reflection and two other reflections that can be attributed to (1 0 0), (1 1 0), and (2 0 0), indicates the hexagonal arrangement of pores [17,18]. This is consistent with the XRD characteristic diffraction reflection of the ordered hexagonal structure of MCM-41 molecular sieve which is reported by Beck, Vartuli et al. [8], indicating that the three catalyst carriers have the ordered hexagonal structure of MCM-41. The reflections that attribute to (1 1 0) and (2 0 0) are less intense in MCM-41 (0.3) and are not present in MCM-41 (0.1). The MCM-41(0.2) has the highest intensity of the reflections compared with other two MCM-41 catalyst carriers, which can be attributed to MCM-41(0.2) itself composed of well-formed MCM-41. It can be concluded that the long-range order of hexagonal structure of MCM-41 can be affected by the condensation of template. When the concentration of template is low, the high concentration of silicate species tends to self-condense to form larger polycondensates, the contacts between these polycondensates and template is weak, which is not conducive to the formation of ordered structures. Conversely, if the concentration of template is high, although the silicate species can interact with the template agents, it hinders the formation of template micelles and the self-polycondensation process of silicate species, thus affecting the long-range order structure of MCM-41.

### 3.5. SEM and TEM Analysis of MCM-41

**Figure 7:**
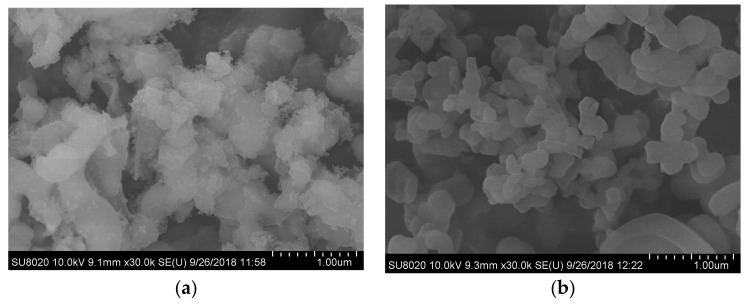

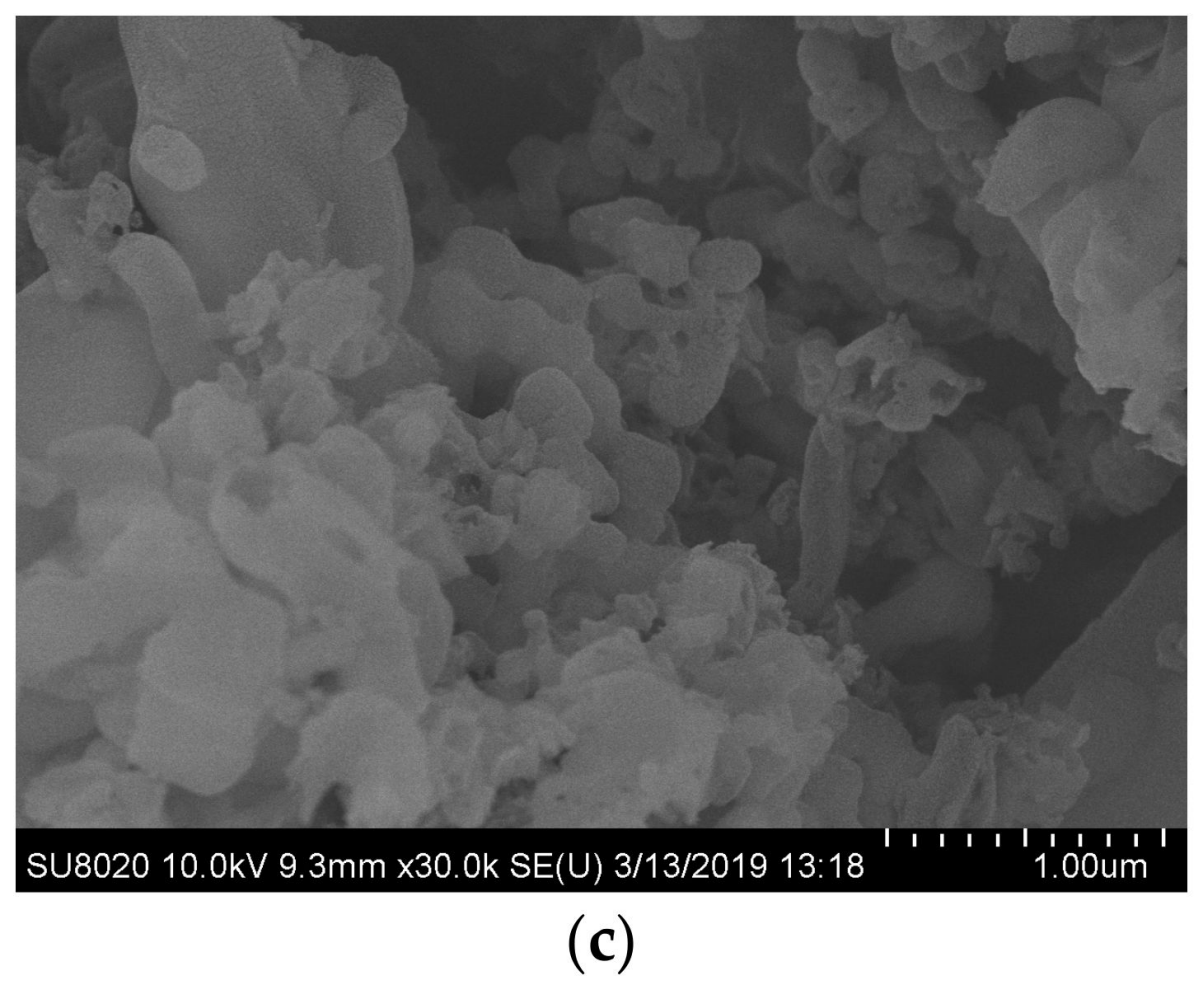
SEM images of MCM-41s: (**a**) MCM-41(0.1); (**b**) MCM-41(0.2); (**c**) MCM-41(0.3).

Figure 7 is the SEM images of MCM-41 catalyst carriers. It can be seen from Figure 7 that three MCM-41 samples are porous materials formed by the accumulation of numerous nano-scale particles, and the particle size is about 300 to 500 nm.

**Figure 8:**
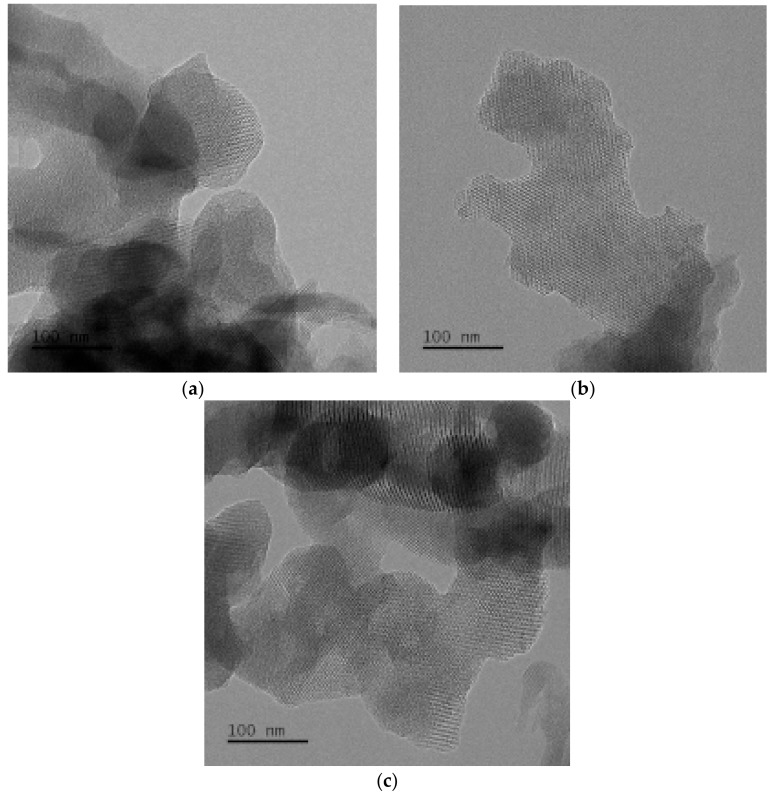
TEM images of MCM-41s: (**a**) MCM-41(0.1); (**b**) MCM-41(0.2); (**c**) MCM-41(0.3).

Figure 8 is the TEM images of MCM-41 catalyst carriers. It can be seen from Figure 8a–c that an ordered hexagonal structure can be observed in the three samples. The mesoporous structure has parallel channels. The longitudinal channel grows along the channel and penetrates the entire grain. In the TEM images of MCM-41(0.1) and MCM-41(0.3), some amorphous substances particles can be observed, which does not contain the characteristic hexagonal structure of MCM-41, combined with XRD and SEM results can be inferred that the amorphous substances particles are amorphous silica. The same particles could not be observed in MCM-41(0.2).

According to the liquid crystal template mechanism originally proposed by the Mobil scientists [8,19], in the synthesis process of molecular sieves the organic template is first dissolved in an aqueous solution, and the hydrophilic groups contact with water and the hydrophobic agents is inwardly aggregating. The aggregations of CTAB surfactant micelle forming into rods are in a hexagonal arrangement in solution. Inorganic silicate present in the mixtures could form around these micellar arrays to produce an inorganic structure, presenting the hexagonal structure. Then the organic template is removed by calcination to obtain MCM-41 molecular sieve with an ordered hexagonal pore structure [20,21].

It can be seen from the results that the concentration of the template has a certain influence on the morphology of MCM-41 molecular sieve. It is due to the fact that when the template in solution is at a lower concentration, and part of inorganic silicate cannot form around micellar arrays, instead they aggregate to form amorphous silica. When the template in solution is at a higher concentration, the template cannot form into ordered hexagonal array structure, which hinders the contact between the silicon species and the template. Combined with XRD analysis results, when n(CTAB):n(Si) = 0.2:1, the MCM-41 molecular sieve have the most orderly structure.

### 3.6. Characterization of Mn-Ce/MCM-41

**Figure 9:**
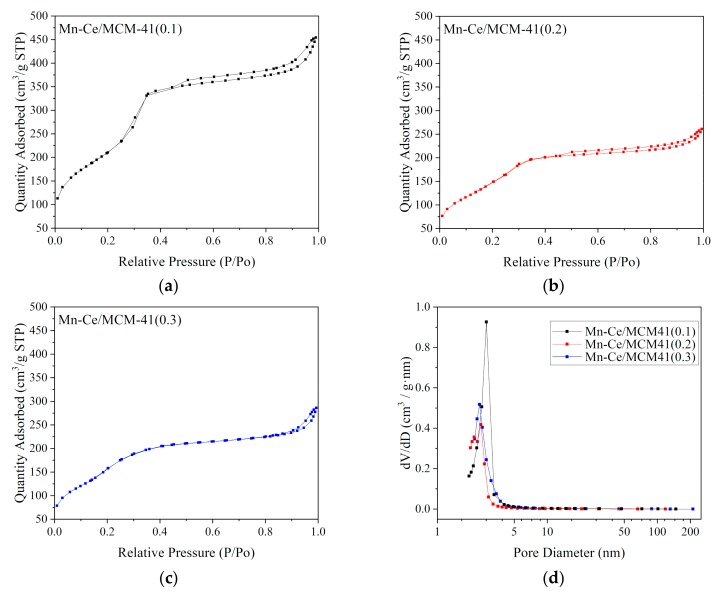
N_2_ adsorption- desorption isotherms (**a**–**c**) and pore diameter distribution curves (**d**) of Mn-Ce/MCM-41(x).

The research of using silica and titanium dioxide as support has been reported by our predecessors in our research team [22]. The chemical compositions of the Mn-Ce/MCM-41 catalysts were measured by XRF and the weight percentage of each elements in the catalysts are listed in Table 4. The results show that Mn-Ce/MCM-41(0.2) contained the highest oxides concentration of Mn and Ce, which indicates that the MCM-41 with more long-range order of hexagonal structure could load more metal oxides during the impregnation. Figure 9 shows the nitrogen adsorption-desorption isotherms (a–c) and the corresponding BJH pore diameter distributions (d) of Mn-Ce/MCM-41(x) catalysts, the pore diameter distributions were derived from BJH adsorption curves. When the metal oxides are loaded, the adsorption and desorption curves of Mn-Ce/MCM-41(0.2) and Mn-Ce/MCM-41(0.3) became reversible and almost coinciding with each other. It indicates that the pores are jammed with the impregnation of metal oxides. The obvious decrease of the specific area as well as the pore volume are shown in Table 4. For Mn-Ce/MCM-41(0.1), the unclosed hysteresis loop at P/P_0_ > 0.8 became obvious, indicating the increase of interparticle pores because of the agglomeration of impregnated metal oxides on the MCM-41 surface.

**Figure 10:**
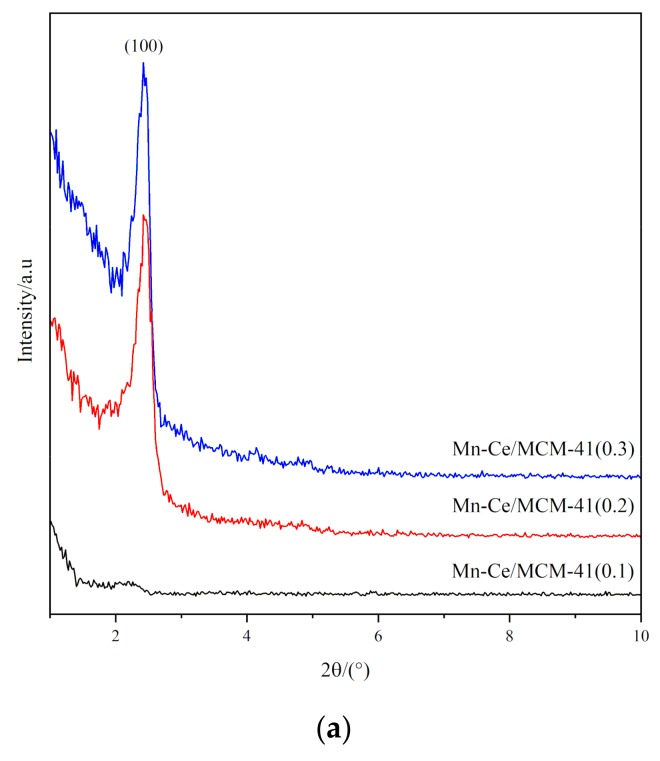

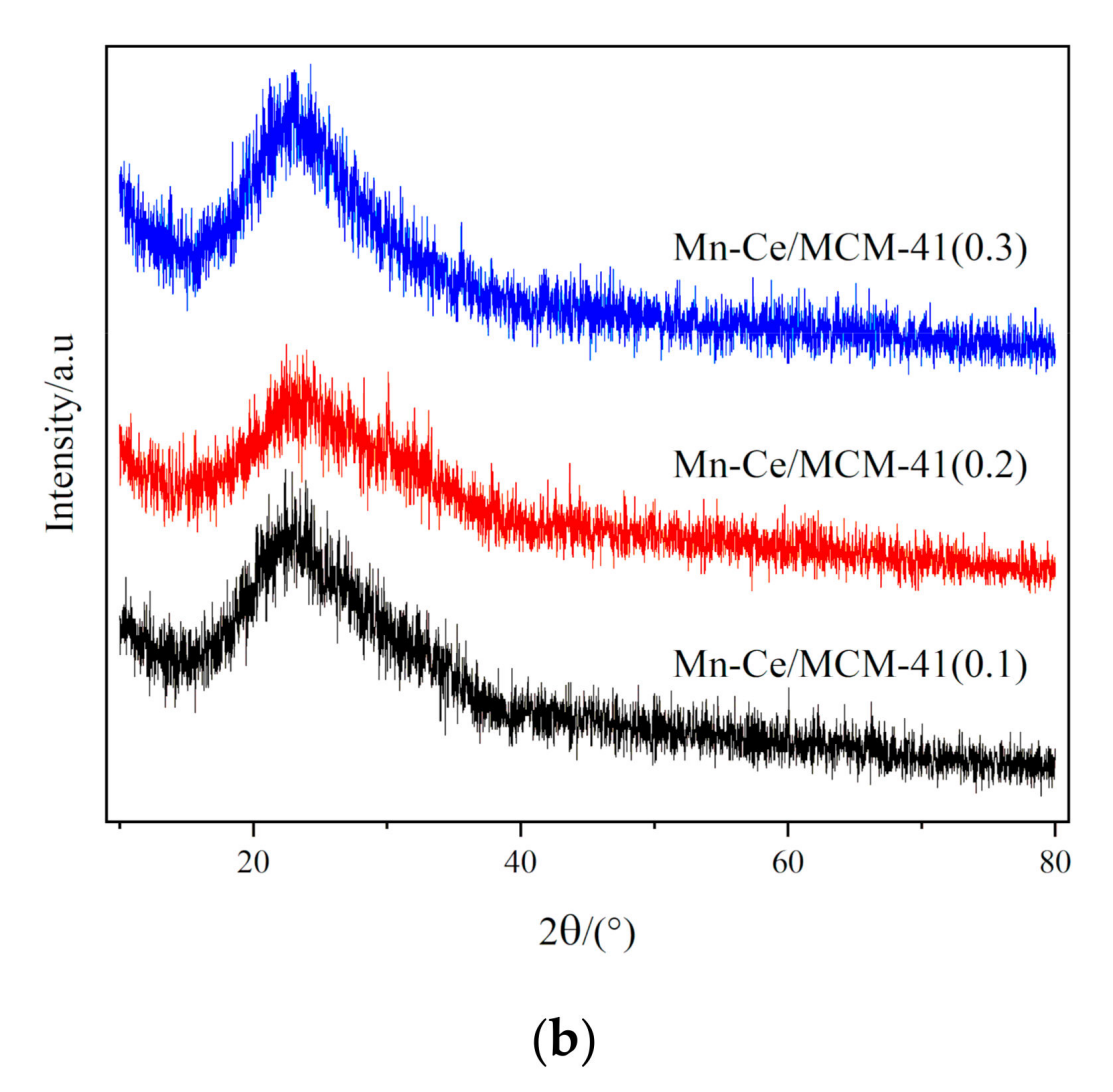
XRD patterns of Mn-Ce/MCM-41(x): (**a**) 2θ = 1–10°; (**b**) 2θ = 10–80°.

The XRD patterns of Mn-Ce/MCM-41 catalysts are shown in Figure 10. Figure 10a is the XRD patterns of Mn-Ce/MCM-41 at low diffraction angle, for Mn-Ce/MCM-41(0.2) and Mn-Ce/MCM-41(0.3), an intense (1 0 0) reflection is clearly observed. However, compared with MCM-41(0.2) and MCM-41(0.3) in Figure 6, the reflections of (1 1 0) and (2 0 0) cannot be found in Figure 10a and the intensity of (1 0 0) reflection in Mn-Ce/MCM-41(0.2) and Mn-Ce/MCM-41(0.3) is decreased. No reflections are observed in Mn-Ce/MCM-41(0.1), indicating that after the impregnation, the pores become smaller and hexagonally order structures are partially destroyed.

Figure 10b is the high angle XRD patterns of Mn-Ce/MCM-41 catalysts. For all samples, a broad reflection at about 2θ ≈ 22° is ascribed to the amorphous silica. Mn-containing or Ce-containing species are not detected, which suggests that they are probably highly disperse or in an amorphous state in the catalysts. The effects of different catalyst supports and synthetic methods on the exact nature of CeO_2_ and MnO_x_ species present on catalyst surface are currently under investigation in our laboratory.

### 3.7. Catalyst Activity of Mn-Ce/MCM-41

**Figure 11:**
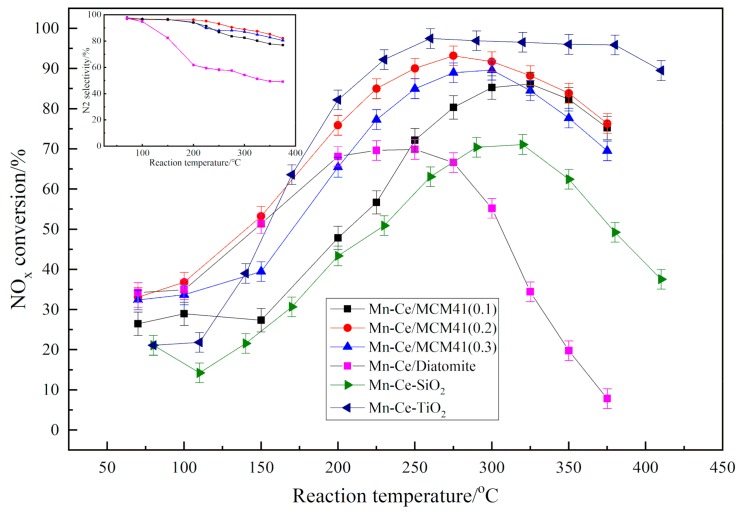
Denitration activity and N_2_ selectivity of catalytic materials.

The research of using silica and titanium dioxide as support has been reported by our predecessors in our research team [22]. As shown in Figure 11, the NO_x_ conversion of Mn-Ce/MCM-41 catalysts have a significantly enhancement compare to Mn-Ce/Diatomite and Mn-Ce-SiO_2_. Although the denitration activity of Mn-Ce/MCM-41(0.2) is slightly lower than the Mn-Ce-TiO_2_, the MCM-41 from diatomite still can be used as a catalytic support, and it has the potential to replace TiO_2_ as a SCR catalyst support. Mn-Ce/MCM-41(0.2) sample exhibits the highest activity, attaining 93% NO_x_ conversion at 275 °C. Mn-Ce/MCM-41 (0.2) catalyst is showing best activity because it has high catalyst loading. However, the Mn-Ce/MCM-41 (0.1) catalyst has 3–4 times lower catalyst loading compared to other Mn-Ce/MCM-41 (0.2 and 0.3), but its denitration activity is not 3–4 times lower. Indicates that the structure of MCM-41 can improve the denitration activity under low active components loading. The NO_x_ conversion of Mn-Ce/MCM-41 catalysts are decreasing with rising temperature to 300 °C, and the N_2_ selectivity began to decrease at 225 °C, this phenomenon is due to the competitive reaction between SCR and NH_3_ oxidation. In the low temperature range, SCR activity increases with temperature, but abundant NO_2_ and NO gases will be generated at higher temperature because of the oxidation of NH_3_. It is worth noting that Mn-Ce/MCM-41(0.2) has the smallest specific surface area among the three catalyst samples, which indicates that the specific surface area is not the most decisive factor affecting denitration activity of the molecular sieve catalyst.

**Figure 12:**
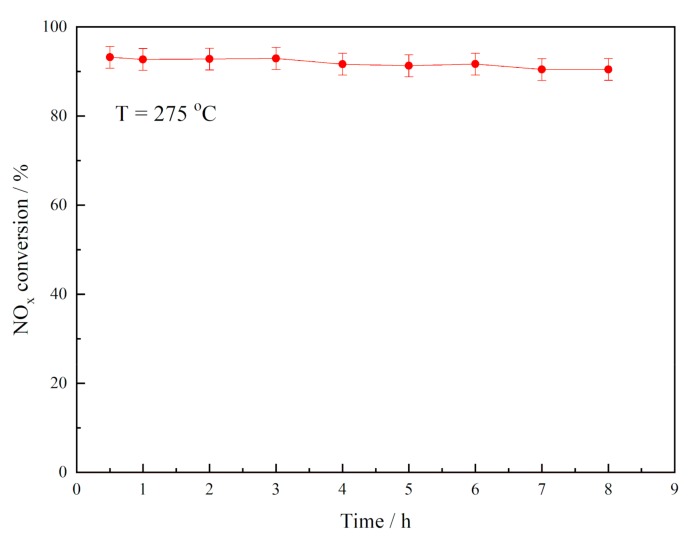
The denitration activity of Mn-Ce/MCM-41(0.2) under continuously mixed gas.

The Mn-Ce/MCM-41(0.2) was tested under the continuously inputted mix gas circumstance at the temperature of 275 °C to study the stability of the catalyst. The result of denitration activity is shown in Figure 12. It can be seen that the denitration activity of Mn-Ce/MCM-41(0.2) did not have an obvious decrease during the 8-hour test, indicating a relatively stability of the catalyst. The service life of the catalyst in practical application still needs a further study.

### 3.8. NH_3_-TPD Analysis of Mn-Ce/MCM-41

**Figure 13:**
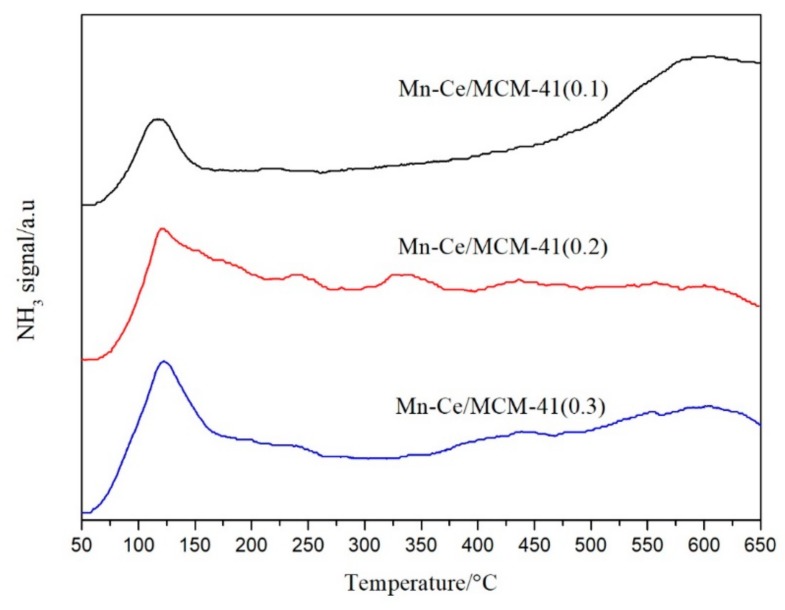
NH_3_-TPD diagram of catalytic materials with different ratio of template.

Temperature programmed desorption of NH_3_ have been carried out to investigate the adsorption of NH_3_ on the catalysts. Because of the limitation of the lab, the acid sites in the catalysts were not normalized by the surface area, the analysis in this section provides only a qualitative comparison of total acid sites in the three catalysts. Figure 13 shows the NH_3_-TPD results of Mn-Ce/MCM-41 catalysts, three catalysts all have a desorption peak of NH_3_ at 50–250 °C, which can be attributed to the adsorption of NH_3_ adsorbed by the weak Lewis acid sites [23,24]. With the increase of temperature, the NH_3_ desorption of Mn-Ce/MCM-41(0.1) gradually became uniform and increase after 400 °C, indicating the presence of strong acid sites in the catalyst [25]. According to the normalized peak area, the relative peak area percentages of Mn-Ce/MCM-41(0.1), Mn-Ce/MCM-41(0.2), and Mn-Ce/MCM-41(0.3) at 50–250 °C are 0.198, 0.345, and 0.325, the relative peak area at 500–650 °C are 0.487, 0.234, and 0.311, which indicates that the NH_3_ desorption of Mn-Ce/MCM-41(0.2) and Mn-Ce/MCM-41(0.3) is enriched in the low temperature area. It can be considered that the more regular MCM-41 hexagonal structure can provide more weak acid sites, and fewer strong acid sites. Mn-Ce/MCM-41(0.2) and Mn-Ce/MCM-41(0.3) has a relatively high NH_3_ desorption peak in the range of 50–250 °C. However, the NH_3_ desorption of Mn-Ce/MCM-41(0.3) gradually is decreasing after 250 °C, while the NH_3_ desorption of Mn-Ce/MCM-41(0.2) is stable at a relatively high level after 250 °C. Therefore, Mn-Ce/MCM-41(0.2) has better low-temperature denitration performance than the other two catalysts.

### 3.9. NH_3_-DRIFTS Analysis of Mn-Ce/MCM-41

**Figure 14:**
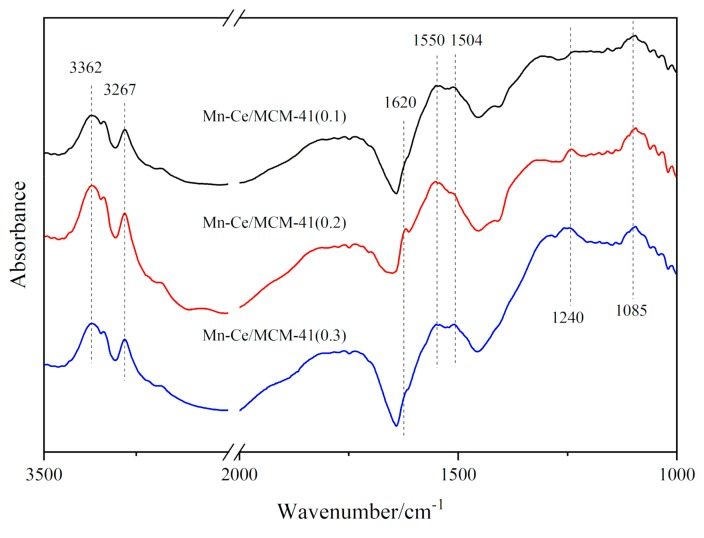
In situ DRIFTS spectra of NH_3_ adsorption over catalytic materials with different ratio of template at 275 °C.

In order to identify the types of surface acid site, in situ DRIFTS measurements of NH_3_ adsorption over catalysts were carried out. In Figure 14, the in situ DRIFTS spectra of adsorbed NH_3_ species over Mn-Ce/MCM-41 surface were obtained after NH_3_ adsorption for 1 h at 275 °C and subsequent purging with N_2_ for 30 min. The bands at 3362, 3267 cm^−1^ correspond to the NH_3_ adsorbed species bonding to Lewis acid sites [26], while the weak bands at 1620, 1143, and 1085 cm^−1^ are attributed to NH_3_ coordinating on Lewis acid sites [27,28]. The band at 1240 cm^−1^ is attributed to the symmetric bending vibration of NH_3_ chemisorbed on Lewis acid sites [27]. NH_2_ species are observed at 1550 and 1504 cm^−1^ [29]. It can be seen that there are a certain number of Lewis acid sites on the surface of the three catalytic materials, while the characteristic peaks left by Mn-Ce/MCM-41(0.2) after adsorbing NH_3_ are relatively strong, indicating that the catalyst surface has the more Lewis acid sites.

## 4. Conclusions

In this paper, diatomite was used as the raw material, cetyltrimethylammonium bromide was used as the template, and MCM-41 molecular sieve with different molar ratio of CTAB to silicon was prepared by hydrothermal method as the carrier, MnO_x_ and CeO_2_ were used as the active components of denitration catalysts. The results show that the denitration efficiency of the MCM-41 molecular sieve catalyst is generally higher than Mn-Ce/diatomite. At the same time, when the molar ratio of CTAB to silicon is n(CTAB):n(Si) = 0.2:1, MCM-41 molecular sieve has the highest specific surface area and high pore volume. Microscopically, the sample is porous materials formed by the accumulation of numerous nano-scale particles, the particle size is about 300 to 500 nm, and a highly ordered hexagonal structure can be observed inside the particle. This indicates that the diatomite can be used as a raw material for the synthesis of MCM-41 molecular sieve. In situ DRIFTS results indicate that the more Lewis acid sites exist on the surface of catalyst, which lead to better low temperature SCR denitration performance, and the denitration efficiency can reach up to 93%. Therefore, the MCM-41 synthesized from diatomite can be used as a catalytic support.

## Figures and Tables

**Table 1:** The SiO_2_ content in diatomite and filter residue.

Sample	Mass (g)	SiO_2_ (%)
Diatomite	10	95.3638
filter residue 1	5.0228	91.1808
filter residue 2	5.1064	90.6944

**Table 2:** The main chemical composition of diatomite.

Component	SiO_2_	Fe_2_O_3_	Al_2_O_3_	K_2_O	CaO	Na_2_O
Content (%)	95.36	1.41	1.08	0.86	0.62	0.22

**Table 3:** Specific surface area and pore structure of diatomite and MCM-41 with different ratio of template.

Sample	S_BET_ (m^2^/g)	Pore Volume (cm^3^/g)	Average Pore Diameter (nm)
MCM-41(0.1)	858.3	0.78	3.4
MCM-41(0.2)	941.8	0.94	3.3
MCM-41(0.3)	924.1	0.96	3.4

**Table 4:** Chemical compositions of catalysts, specific surface area and pore structure of Mn-Ce/MCM-41 catalysts.

Catalyst	Chemical Compositions (wt.%)			S_BET_ (m^2^/g)	Pore Volume (cm^3^/g)	Average Pore Diameter (nm)
	MnO	CeO_2_	SiO_2_			
Mn-Ce/MCM-41(0.1)	3.73	2.58	93.46	829.3	0.78	3.3
Mn-Ce/MCM-41(0.2)	13.28	6.65	79.75	587.1	0.48	3.3
Mn-Ce/MCM-41(0.3)	9.29	5.30	85.13	615.1	0.52	3.4
Mn-Ce/Diatomite	2.38	2.22	90.93	16.9	0.06	11.5
Mn-Ce-SiO_2_ [22]	2.68	1.56	95.47	267.9	0.60	8.7
Mn-Ce-TiO_2_ [22]	MnO	CeO_2_	TiO_2_	124.9	0.17	4.2
	17.32	8.79	62.03			
